# Recent Experimental Studies of Maternal Obesity, Diabetes during Pregnancy and the Developmental Origins of Cardiovascular Disease

**DOI:** 10.3390/ijms23084467

**Published:** 2022-04-18

**Authors:** Stephanie M. Kereliuk, Vernon W. Dolinsky

**Affiliations:** 1 Diabetes Research Envisioned and Accomplished in Manitoba (DREAM) Research Theme of the Children’s Hospital Research Institute of Manitoba, 715 McDermot Avenue, Winnipeg, MB R3E 3P4, Canada; umkereli@myumanitoba.ca; 2 Department of Pharmacology and Therapeutics, University of Manitoba, Winnipeg, MB R3E 0T6, Canada

**Keywords:** cardiovascular disease, developmental programming, diabetes, gestational diabetes, maternal obesity, pregnancy

## Abstract

Globally, cardiovascular disease remains the leading cause of death. Most concerning is the rise in cardiovascular risk factors including obesity, diabetes and hypertension among youth, which increases the likelihood of the development of earlier and more severe cardiovascular disease. While lifestyle factors are involved in these trends, an increasing body of evidence implicates environmental exposures in early life on health outcomes in adulthood. Maternal obesity and diabetes during pregnancy, which have increased dramatically in recent years, also have profound effects on fetal growth and development. Mounting evidence is emerging that maternal obesity and diabetes during pregnancy have lifelong effects on cardiovascular risk factors and heart disease development. However, the mechanisms responsible for these observations are unknown. In this review, we summarize the findings of recent experimental studies, showing that maternal obesity and diabetes during pregnancy affect energy metabolism and heart disease development in the offspring, with a focus on the mechanisms involved. We also evaluate early proof-of-concept studies for interventions that could mitigate maternal obesity and gestational diabetes-induced cardiovascular disease risk in the offspring.

## 1. Introduction

Globally, cardiovascular disease (CVD) is a major cause of all-cause mortality. Several well-recognized risk factors exist, including hypertension, hyperlipidemia, obesity and diabetes. Evidence from the population level, clinical cohorts and animal models identified the influence of the early life environment on CVD development in the offspring. The mechanisms responsible for the effects of the early life environment are only beginning to emerge, but this knowledge raises the interesting possibility that earlier interventions could reduce the incidence of CVD. In our review, we summarize evidence that examined how maternal obesity and diabetes during pregnancy affect CVD development in the offspring, with a focus on the mechanisms these studies identified. We also evaluate emerging interventional studies focused on mitigating maternal obesity and gestational diabetes-induced CVD risk in the offspring.

## 2. Cardiovascular Disease and Diabetes

In 2020, the World Health Organization estimated that 17.9 million deaths were a consequence of CVD, making it the deadliest non-communicable disease worldwide. CVDs are a group of complex chronic disorders affecting the heart and blood vessels. CVDs include ischemic heart disease (coronary artery disease which manifests acutely as a myocardial infarction), heart failure, arrhythmias and cerebrovascular disease, among others [1]. Complex pathophysiological processes are involved in CVD development. Initially, CVD is perceived to begin as an adaptive response by the heart to stress that becomes maladaptive as the disease advances. Over its 74-year length, the Framingham Heart Study has demonstrated that hypertension, coronary artery disease, cardiac hypertension and diabetes are cardiovascular risk factors associated with the future development of heart failure [2,3].

According to the International Diabetes Federation, approximately 463 million people currently live with diabetes, and the incidence is continuing to rise; current projections estimate that a startling 700 million individuals will have diabetes by 2045 [4]. Destruction of the insulin-producing pancreatic beta cells due to an immune-associated or environmental insult results in type 1 diabetes [5]. Individuals with type 1 diabetes have a ten-times-greater risk of cardiovascular events than age-matched populations without diabetes [6]. However, the majority of the individuals living with diabetes have type 2 diabetes (T2D), and the rapid rise in diabetes incidence is driven by the increase in T2D. In T2D, hyperglycemia is generally viewed to be a consequence of peripheral tissue of insulin resistance and insufficient insulin secretion, though T2D is a heterogenous disorder [7]. In T2D, hyperinsulinemia is frequently featured because insulin target tissues are unable to perform glucose-lowering responses at normal plasma insulin levels, resulting in increased insulin secretion to compensate [8,9]. Obesity is a key risk factor for the development of T2D by promoting insulin resistance [7,10]. These trends are serious public health concerns because diabetes is a systemic disorder. Epidemiological evidence shows that diabetes is an independent risk factor for the development of CVD [7,11,12,13,14,15,16], and diabetes-related morbidity and mortality is primarily due to cardiovascular complications [17,18,19].

## 3. Youth Onset Type 2 Diabetes and Cardiovascular Disease

The rising rates of obesity and T2D in adolescents put these youth at greater risk of CVD as they age [20,21,22]. Many groups have established that hypertension and obesity in childhood result in poor cardiovascular outcomes with age [23,24,25,26,27]. These findings are striking, considering that the Framingham Heart Study was begun in 1948 to identify factors for CVD in the general population [28] and has now evolved to include the children and grandchildren from the original cohort, as well as two additional minority cohorts, to assess temporal trends in CVD development and investigate the genetic architecture of CVD and its risk factors [3,29,30].

Using this approach, parental history of coronary artery disease and parental death by coronary artery disease were observed to be significant independent predictors of offspring coronary artery disease [31]. Subsequently, parental heart failure was shown to be associated with a greater risk of LV systolic dysfunction and heart failure in the offspring [32]. Furthermore, the Framingham study also showed that maternal and paternal diabetes conferred equivalent risk for offspring T2D; however, maternal diabetes conferred a greater risk of abnormal glucose tolerance in the offspring, when compared to paternal diabetes [33]. Recently, Mendelson et al. demonstrated that maternal pre-pregnancy LDL cholesterol levels were associated with adult offspring low-density lipoprotein (LDL) cholesterol levels after adjustments for offspring lifestyle and inherited genetic variants [34]. Exposure to elevated pre-pregnancy LDL cholesterol levels increased the risk for having elevated LDL cholesterol levels 3.8 times in adult offspring [34]. These findings support the likelihood for parental transmission of cardiometabolic disease risk factors and a possible epigenetic contribution to CVD risk in the general population that merits further investigation [34].

**Figure 1:**
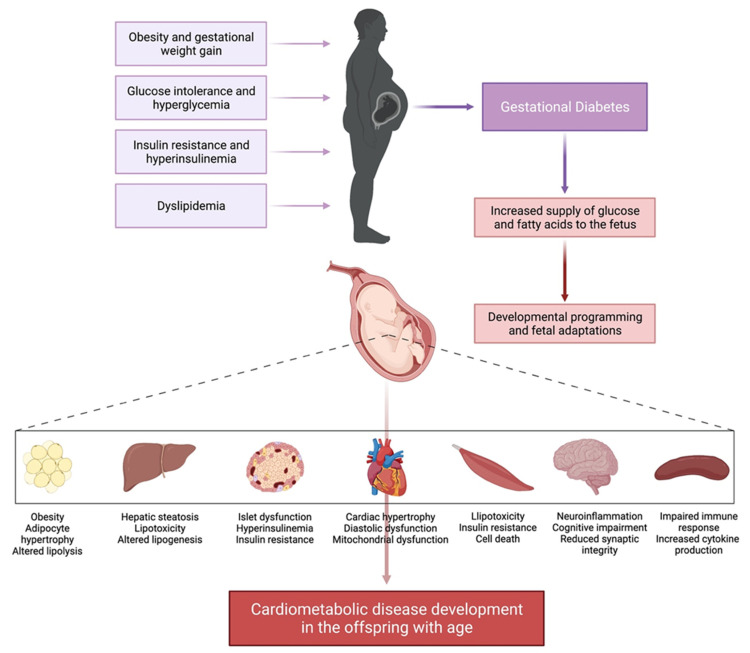
Influence of maternal obesity and gestational diabetes mellitus (GDM) on the developmental origins of cardiometabolic disease in the offspring. Obesity, dysglycemia and hyperlipidemia pre-pregnancy and during pregnancy contribute to the development of GDM. GDM increases the supply of glucose and fatty acids to the fetus which results in fetal adaptations and developmental programming that include alterations in gene expression, organogenesis, cellular metabolism and epigenetic modifications. GDM exposure appears to affect a wide range of cellular, tissue and organ systems in a manner that increases the risk of cardiometabolic disease in the offspring with increasing evidence from rodent model systems that the cardiovascular system is sensitive to GDM-induced effects. Created with BioRender.com.

Overall, these findings suggest that factors such as the early life environment could be at play driving CVD and cardiometabolic disease among youth. Thus, the central focus of this review will be to summarize recent evidence showing that diabetes during pregnancy and maternal obesity affects the development of CVD (Figure 1).

## 4. The Developmental Origins of Health and Disease Theory

Traditionally, the development of chronic disease in adulthood was attributed to the interaction between genetic influences and lifestyle factors. The heart is the first functional organ to develop in the mammalian embryo. Cardiogenesis begins on embryonic day 6.5 in mice, 8.5 in rats and 18–19 in humans. The heart begins to beat and pump blood, its primary function, on embryonic day 8 in mice, 10 in rats and 22 in humans [35]. Therefore, it is a logical hypothesis that early life events that occur during fetal development could induce long-term effects on the structure, function and metabolism in heart.

The collective evidence from epidemiological studies, clinical cohorts and animal model experiments supports the theory that environmental exposures in early life can determine the long-term health of individuals, including cardiovascular and metabolic diseases [36,37]. Dr. David Barker was the first to propose the developmental origins of health and disease (DOHaD) theory. Barker’s foundational work identified associations between undernutrition during gestation, low birth weight and higher rates of ischemic heart and coronary artery disease mortality in adulthood in British children born in the 1920s [38,39,40,41]. Undernutrition and low birth weight was also associated with insulin resistance in this population later in life [42]. The DOHaD theory was further refined through additional population-based birth cohort studies, such as the Dutch famine and Leningrad siege studies, that supported the effect of undernutrition on CVD development (reviewed in Agarwal et al. [37]). Thus, the DOHaD theory proposed that nutrient restriction during specific periods of development (e.g., conception, gestation and the first few years of life), when tissues and organs are particularly sensitive to environmental exposures, as a consequence influence the susceptibility for cardiovascular and metabolic diseases in adulthood [41,42,43].

Evidence for the DOHaD theory was initially based on evidence from low birth weight and maternal undernutrition; however, in the developed world, caloric excess, obesity and diabetes during pregnancy are more prevalent. Evidence is mounting that altered fetal growth and development, as well as lifelong perturbations in metabolic tissues, are also associated with exposure to overnutrition and metabolic disorders in early life (reviewed in [37,44,45]). Human DOHaD research has been conducted in prospective mother-offspring cohort studies [46,47]. Due to limitations associated with longitudinal follow-up, few cohort studies have reached middle age when major CVD endpoints are available. Moreover, the multifaceted nature of early life exposures and the interaction with the postnatal environment creates difficulties to establish the specific impact of one exposure on CVD health outcomes in the offspring in a prospective mother-offspring study. Despite these confounding factors, the controlled environment in animal model experiments is useful to confirm the independent impact of prenatal exposures and address how these impact the future cardiovascular health of the offspring. However, it remains essential to replicate animal model findings in human populations.

## 5. Maternal Obesity and Gestational Diabetes Mellitus

As the rates of obesity continue to rise in the general population, women are frequently reaching childbearing age obese, which is a key risk factor for the development of gestational diabetes mellitus (GDM) [48,49]. It has also been reported that impaired fasting blood glucose, elevated fasting insulin and dyslipidemia prior to pregnancy are associated with GDM, and in the Coronary Artery Risk Development in Young Adults (CARDIA) study, 26.7% of overweight women with one or more cardiometabolic disease risk factors developed GDM compared to 7.4% with no risk factors [50]. These observations imply that cardiometabolic impairments exist in nondiabetic women prior to pregnancy and can indicate risk for future GDM development.

GDM is defined as glucose intolerance and hyperglycemia with first onset during pregnancy [51,52,53]. The International Diabetes Federation estimates that one in six of the annual 20 million live births are affected by hyperglycemia, with 16% related to diabetes during pregnancy and the remaining 84% related to GDM, making it the most common complication of pregnancy [4]. GDM is typically diagnosed in the third trimester after administration of an oral glucose tolerance test at 24–28 weeks of gestation [54]. GDM is characterized by hyperglycemia, insulin resistance and increased gestational weight gain. A healthy pregnancy is associated with increasing insulin resistance (in order to spare more glucose for the fetus). In GDM, β-cell insulin secretion is not sufficient to compensate for the insulin resistance of pregnancy, resulting in hyperglycemia during pregnancy [55]. In women diagnosed with GDM, blood glucose levels return to normal following delivery [54], albeit with a greater risk of T2D [56,57,58] as well as CVD events and mortality as they age [59,60,61]. In women with GDM, the increased risk of CVD development occurs independently and in addition to the risk attributed to T2D development [62,63].

While GDM imparts significant cardiometabolic risk to the mother, it also increases the risk for cardiometabolic and CVD development in the offspring [64]. GDM is associated with greater incidence of large-for-gestational-age infants, fetal adiposity and macrosomia, neonatal hyperinsulinemia and shoulder dystocia, and increased risk of preterm delivery [65,66,67,68,69]. Hyperglycemia throughout gestation is associated with significant negative long-term effects on the health of the offspring [64,66]. Diabetes during pregnancy also predisposes offspring for the development of obesity, T2D and cardiometabolic disease risk factors [33,70,71,72,73,74,75,76,77] (Figure 1). Fetal hypertrophic cardiomyopathy is a well-recognized feature of GDM, though it was believed that this resolved within a few months of birth [78,79,80,81,82]. A limited number of studies linked GDM exposure to increased CVD risk in the offspring [73,83,84,85,86]. Serial echocardiography of fetuses from diabetes during pregnancy revealed increased LV wall and intraventricular septal wall thickness [87]. Postnatal echocardiography at 6 weeks and 12 months of age, and subsequent follow-up at 3–8 years of age, showed that cardiac hypertrophy persisted long after the intrauterine exposure to hyperglycemia was removed [86,87]. The persistence of increased LV wall thickness into early childhood suggests that additional factors beyond glycemic control and growth factors are involved and gives credence to the concept of fetal programming of cardiovascular health. Therefore, GDM has long-term implications for both maternal and offspring cardiovascular health.

## 6. Animal Models of Maternal Obesity and Diabetes in Pregnancy

While human studies are important to the field, it is difficult to control for confounding factors over a lengthy and expensive study period, and genetic variability adds an additional level of complexity. Thus, animal models with a common genetic background and carefully controlled dietary and activity conditions are useful for examining the long-term cardiovascular effects of early life exposure to maternal obesity and diabetes during pregnancy, as well as determining mechanisms involved. There are also limitations inherent to using animals to model human pregnancy. These include differences in placental morphology, gestational length, parturition, a greater number of offspring per pregnancy, and different developmental windows of fetal and neonatal cellular differentiation and organogenesis [88,89]. We recently reviewed the effects of maternal obesity and diabetes during pregnancy on metabolic health outcomes in the offspring [37]. Therefore, we begin by describing some of the models used and briefly report their effects on cardiometabolic disease development (Figure 1). Then we provide a detailed review of studies that examined CVD development in the offspring and the mechanisms involved.

### 6.1. Metabolic Disease Development in Offspring Exposed to Diabetes during Pregnancy and Maternal Obesity

Recent studies have characterized the influence of maternal obesity and diabetes during pregnancy on CVD risk factors, including obesity, hyperlipidemia and insulin resistance in young adult rodent offspring (Figure 1).

To model diabetes during pregnancy, streptozotocin (STZ) has been used to cause β-cell destruction in order to produce maternal insulin deficiency and hyperglycemia, though this more closely approximates type 1 diabetes [90,91,92]. Despite maternal plasma glucose levels exceeding the clinical definition of GDM, 80-day-old Wistar rat offspring [90] and 15-week-old Sprague-Dawley rat offspring [92] remained glucose tolerant with normal islet morphology [92] and retained similar plasma glucose and insulin levels as controls [90]. The majority of women experiencing diabetes during pregnancy do not exhibit insulin deficiency but are often hyperinsulinemic and do not display excessive hyperglycemia, both of which occur following STZ administration. Consequently, insulin deficiency using STZ may not accurately model the mechanisms involving GDM and its effects on the offspring cardiovascular system [88].

A common approach to model maternal obesity involves feeding animals diets that are high in a combination of saturated fats and simple sugars. A number of groups have fed female rodents a high-fat diet supplemented with sweetened condensed milk to cause obesity before, during and in the post-pregnancy period, and then compare them to controls fed a chow diet [93,94]. These pregnant, obese mice were hyperinsulinemic, but without hyperglycemia [94]. At weaning (21 days after birth), these obese dams were both hyperglycemic and hyperinsulinemic, compared to standard chow-fed dams [94].

Using this approach, a number of studies showed that maternal obesity induced greater adiposity and obesity [95,96,97,98] as well as hepatic steatosis [95,96,99] in the offspring. Several of these studies have reported hyperlipidemia [96,100], including elevated free fatty acids, plasma triacylglycerol and reduced total and HDL cholesterol in the offspring, though one study did not observe alterations in plasma lipids [101]. Reduced insulin signalling was apparent in the adipose tissues and vastus lateralis muscle of the offspring, consistent with a reduction in insulin sensitivity [98].

Our group showed that compared to low-fat-fed control females, feeding female Sprague-Dawley rats a high-fat-and-sucrose diet prior to mating and throughout pregnancy increased pre-pregnancy weight, and then during pregnancy caused excessive gestational weight gain, hyperglycemia and hyperinsulinemia and glucose intolerance at mid-gestation that are characteristic of GDM [102]. Using this model of diet-induced GDM, we determined how the interaction between prenatal GDM exposure and the offspring postnatal diet affected health outcomes in the offspring. At weaning, the offspring of GDM dams and the lean controls were randomly assigned to a low-fat or high-fat-and-sucrose diet [102,103,104]. Using this experimental protocol, we demonstrated that GDM exposure conditioned 3-month-old offspring to the development of obesity, hepatic steatosis, glucose intolerance [102] and pancreatic islet dysfunction [103,104] (Figure 1). GDM exposure and postnatal high-fat-and-sucrose diet consumption additively increased offspring body weight compared to independent GDM exposure or postnatal high-fat diet feeding. Hepatic steatosis in the GDM exposed offspring was associated with an altered liver metabolome that was characterized by diacylglycerol accumulation and reduced phosphatidylethanolamine lipid species [102]. Diacylglycerol is a lipotoxic lipid and cellular secondary messenger, while phosphatidylethanolamine is a membrane phospholipid. Gene expression analysis revealed a mechanism whereby reduced cytidine triphosphate phosphoethanolamine cytidylyltransferase expression (the rate limiting enzyme for phosphatidylethanolamine synthesis) reduced the utilization of diacylglycerols for phosphatidylethanolamine synthesis and ultimately resulted in diacylglycerol and triacylglycerol accumulation in the livers from young adult offspring of GDM dams [102].

Subsequent studies using this approach have demonstrated that offspring exposed to GDM exhibited skeletal muscle lipotoxicity and insulin resistance [105,106] (Figure 1). Metabolomic profiling of soleus muscle from the rat offspring exposed to GDM revealed the accumulation of diacylglycerols and alterations of the fatty acyl composition of phosphatidic acid and cardiolipin [106]. This was associated with activation of Protein Kinase C (PKC)-δ [106] which inhibits insulin signalling in muscle tissue [107]. Mechanistically, PKC-δ inhibited the expression of miR-133a, which elevated expression of its target, Nix, a mitophagy and cell-death-modulating protein [106]. Consequently, mitochondrial dysfunction and impaired cell death pathways downstream contribute to insulin resistance in the muscle tissue of GDM-exposed offspring [105,106]. Further studies using this GDM model have demonstrated that the offspring exhibit a hyperactive immune response [108] that manifests in neuroinflammation, which was correlated with cognitive impairment and reduced synaptic integrity [109]. Taken together, these findings demonstrate that exposure to GDM induced obesogenic, lipotoxic and inflammatory conditions in the young adult rat offspring across a spectrum of metabolic tissues (Figure 1).

Another approach to model diabetes during pregnancy has been to combine high-fat-diet-induced maternal obesity (starting four weeks prior to breeding) with late gestation STZ injection (gestational day 14) to cause insulin deficiency, with the intent of causing late-in-gestation hyperglycemia while avoiding glucose-mediated structural heart defects in early organogenesis [110]. In this experimental protocol, insulin was administered to maintain maternal blood glucose between 11.1 and 22.2 mmol/L and minimize ketosis. The combination of obesity and hyperglycemia during pregnancy and its effects on the long-term health of the offspring from birth until 12 months of age was examined. Using this model, body weights as well as serum glucose and triacylglycerol levels were similar to the respective male and female offspring of controls [111]. However, spontaneous mortality was significantly higher, indicating possible confounding effects of exposure to diabetes during pregnancy on the development of cardiac birth defects.

### 6.2. The Effects of Maternal Obesity on Heart Disease Development in The Offspring

Several studies have now examined how maternal obesity affected the cardiovascular health of the offspring. For example, the Ozanne group showed that young adult male offspring (at 3 and 8 weeks of age) from obese dams have increased heart weight, LV wall thickness and increased heart weight to body weight ratio, compared to offspring from control dams [93,112]. Interestingly, at 12 weeks of age, heart weights were not different, consistent with no differences in body weight, adiposity or circulating lipids [112]. Moreover, the effect of maternal obesity on cellular cardiomyocyte hypertrophy were only observed in the 3- and 8-week-old offspring [93,112] and not 12-week-old offspring [112]. In line with cardiac hypertrophy, increased expression of *Nppb*, *Acta1* and the ratio of *Myh7:Myh6* gene expression were observed in the offspring of obese dams [93,112]. However, Louwagie et al. [111] found that maternal high-fat diet feeding increased the heart weight:body weight ratio at 12 months of age in male, but not female offspring.

Maternal obesity reduced LV-developed pressure and increased LV end diastolic volume using Langendorff heart perfusion, revealing the presence of diastolic dysfunction in the offspring [112]. Examination of proteins that regulate the contractile properties of the heart revealed that maternal obesity increased the expression of β1-adrenergic receptors and reduced the expression of troponin and sarcoplasmic/endoplasmic reticulum calcium transporter-2a (SERCA2a) [112]. These findings suggest that maternal obesity has transient effects on cardiac hypertrophy in the offspring, but persistent effects on diastolic function of the LV. This was also consistent with elevated fibrosis observed in the offspring of obese dams [113].

Using this model, a more recent study examined the interaction between maternal obesity and feeding a postnatal high-fat diet to the offspring up to 8 weeks of age. High-fat diet feeding had similar effects on increasing body weight in offspring from obese dams as well as control dams [114]. While maternal obesity and the postnatal high-fat diet independently promoted cardiac hypertrophy and systolic dysfunction, these effects were not additive. However, this study did not evaluate the diastolic function of the heart. A separate publication has shown that maternal high-fat feeding had no effect on systolic or diastolic function of the heart in 12-month-old male and female offspring [111]. Nonetheless, the combination of maternal obesity exposure and postnatal high-fat diet consumption by the offspring did result in enhanced cardiac fibrosis.

At the level of cardiac metabolism, maternal obesity induced significant changes in the serum and cardiac lipidome of e18.5 fetuses that for the most part correlated with the lipid profile of the dams. However, increases in 14:0, 14:1 and 17:0 fatty acids and a decrease in 22:5 fatty acid in the serum of fetal offspring of obese dams diverged from the maternal profile [115]. Maternal obesity significantly increased the abundance of cardiac sphingomyelin in female offspring and reduced cholesterol ester levels in male and female offspring. Interestingly, most triacylglycerols were more abundant while most phosphatidylcholines and odd chain phosphatidylethanolamine phospholipids were less abundant in the hearts of male and female offspring of obese dams. Changes in the overall fatty acid composition of the phospholipids were not observed. In addition, acylcarnitine by-products of fatty acid oxidation were altered in the hearts of these offspring. Hydroxylated acylcarnitines were increased, as were C12:0 and C12:1 acylcarnitines in response to maternal obesity whereas C20:0 and C22:5 were lower in female offspring and in males, C11:0 and C15:0 were higher and C3:0 acylcarnitine was reduced. Cardiomyocytes from female fetuses of obese dams oxidized more 18:1 fatty acid than cardiomyocytes from controls [115]. Changes in lipid species and acylcarnitines could reflect changes in cardiac gene expression. Transcriptomic analysis of the fetal hearts of male offspring of obese dams showed altered expression of PPAR-α and HIF1α regulated genes. Increased expression of *Ppara Cpt1*, *Cpt2* and *Acot2* in both male and female offspring of obese dams was validated by qPCR [115], consistent with increased fat oxidation.

In 8-week-old offspring of obese dams, the expression of insulin receptor-β, a key component for mediating insulin signalling in the heart, was reduced [93]. Interestingly, phosphorylation of Akt at the activating Ser-473 site was elevated in hearts of offspring from obese dams, although this appeared to be mediated by increased p38-Mitogen-activated protein kinase signalling through Erk and mTOR [93], suggesting that protein translation supporting pathologic cardiac hypertrophy was activated.

Reduced levels of mitochondrial localized superoxide dismutase-2 in the hearts of offspring of obese dams [93] suggest that maternal obesity increases the sensitivity of the offspring myocardium to oxidative damage. Maternal high-fat feeding also reduces mitochondrial fission and fusion events in offspring cardiomyocytes, suggesting that there are impairments in the removal of damaged mitochondria [116]. Interestingly, Fernandez-Twinn et al. [93] discovered that exposure of the offspring to maternal obesity increased the cardiac expression of miR-133 and reduced the mRNA expression of one of its putative targets, *Gata-4*, which is upregulated in cardiac hypertrophy.

### 6.3. The Effects of Diabetes during Pregnancy on Heart Disease Development in the Offspring

Using a model of STZ-induced insulin-deficient diabetes during pregnancy, Nakano et al. demonstrated that hyperglycemia promotes the proliferation of fetal offspring cardiomyocytes while maturation was inhibited [117]. Maternal hyperglycemia also reprogrammed cardiac gene expression in fetal rat offspring, with genes involved in myocardial growth, contraction and metabolism most prominently affected [118]. Maternal diabetes also increased cardiomyocyte size and cardiac fibrosis in the offspring [119]. However, maternal diabetes did not affect the LV mass in 12-month-old offspring, though sex-specific effects on cardiac function were observed [111]. Male offspring exhibited reduced diastolic function without systolic impairments, while female offspring exhibited systolic dysfunction, but not diastolic dysfunction, compared to controls [111]. Significant increases in ischemia-induced cardiac infarction have also been reported in adult male and female offspring of STZ-induced diabetes dams [120,121]. Mitochondrial respiration was examined in permeabilized cardiomyocytes and lower basal respiration was observed, but also a higher response to a complex IV substrate, indicating a lower flow of electrons through complexes I-III, but the ability to directly oxidize complex IV substrates, which requires more oxygen [111]. Mitochondrial fission and fusion events were reduced in fetal cardiomyocytes from the STZ-induced diabetes dams, although the ratio of mitochondrial fission was greater than mitochondrial fusion [116]. Baseline levels of mitolysosomes were also elevated [111]. Notably, enhanced expression of autophagy-related proteins Atg5, LC3 II/LC3 I have also been reported [120]. Some of these observations could be a consequence of reduced SIRT1 expression in the hearts from offspring of STZ-induced diabetes dams that is associated with hypermethylation of the *Sirt1* gene [120]. STZ-induced diabetes during pregnancy was also found to stimulate cardiomyocyte apoptosis in the offspring heart via activation of MST1 and downregulation of YAP1 and Survivin [122].

In pregnant rat females fed a high-fat diet in conjunction with STZ-induced diabetes, newborn rat offspring cardiac contractility and LV diastolic functional parameters were impaired as assessed by in vivo echocardiography [110]. Cardiac morphology and function were also examined in these rat offspring aged up to 12 months of age. While LV hypertrophy was not observed in these offspring compared to controls, female offspring exhibited some systolic dysfunction (e.g., reduced ejection fraction) without diastolic dysfunction [111]. In contrast, the male offspring had only diastolic dysfunction (e.g., reduced E/A ratio) and no difference in systolic function.

Despite increased mitochondrial DNA copy numbers in this model [111], these authors found that mitochondria dysfunction was a key feature in the offspring heart. Mitochondrial function was examined across the lifespan in this model. Impaired glucose oxidation that was observed in neonatal cardiomyocytes was attenuated by 10 weeks of age, although reduced respiratory capacity re-emerges in cardiomyocytes at 6 months of age and is present in the cardiomyocytes from 12 months old offspring. In addition, reduced palmitate oxidation and glycolysis were apparent in the neonatal cardiomyocytes. While basal respiration in permeabilized cardiomyocytes from neonatal and 12-month-old offspring were similar, altered state 3 respiration was apparent in mitochondria from 12-month-old offspring, particularly when using complex I fuels as substrates [111]. No differences in mitochondrial complex expression were observed in 12-month-old male offspring hearts, but females had increased protein expression of NDUFA2 (complex I). Fetal cardiomyocytes demonstrated well-balanced fusion and fission events. However, in cardiomyocytes from the male offspring from a high-fat diet and diabetes during pregnancy, mitochondrial fusion and fission events were reduced, but the balance was tipped in favour of more fission events over fusion events [116]. These offspring also have higher baseline levels of mitolysosomes [111]. Morphologically, these mitochondria were shorter, wider and more fragmented. Notably, mitochondrial dynamics in cardiomyocytes from the female offspring were not affected by exposure to maternal diabetes, likely as a consequence of higher protein expression of Mitofusin-2 and OPA1 that could protect against mitophagy-mediated cell death.

In parallel with these metabolic studies, microarray mRNA expression profiling of the hearts of newborn rats was performed. A total of 122 genes were significantly reduced while 201 genes were increased. In agreement with the findings regarding mitochondrial function described above, 32 mitochondrial genes were altered in the offspring of STZ-induced diabetes dams fed a high-fat diet [123]. Several pathways were affected, but functional analysis revealed that downregulation of fibroblast growth factor (FGF) and PI-3-kinase pathways and upregulation of peroxisome proliferator-activated receptor coactivator alpha (PGC1α) mitochondrial biogenesis pathway featured prominently in the offspring heart. Increased PGC1α protein levels and reduced levels FGF receptor-2 were confirmed in the hearts of the offspring, although levels of phosphorylated Akt were similar [123]. These changes could be a consequence of alterations in epigenetic marks that regulate gene expression. For example, chromatin-immunoprecipitation sequencing revealed increases in the gene-activating mark of histone-3 trimethylated lysine 4, but also the gene-repressive histone 3 trimethylated lysine-27 mark [124]. Similar to the transcriptomic profile, genes involved in metabolic processes were highly represented.

### 6.4. The Effects of Maternal Obesity on Vascular Function in The Offspring

A recent systematic review of 17 studies of mother-offspring pairs reported a positive association between higher maternal pre-pregnancy body mass index and increased blood pressure in the offspring, independent from the body mass index of the offspring [125]. Several studies have reported elevated blood pressure as well as vascular remodeling and impaired vascular function between three and six months of age in arteries isolated from rat offspring of obese dams [94,100,126,127]. The combination of exposure to maternal obesity and a postnatal high-fat diet also markedly increased systolic blood pressure in mouse offspring [114]. Moreover, in macaques, maternal high-fat diet, followed by a post-weaning high-fat diet, induced greater vascular remodeling and inflammation than postnatal high-fat feeding of the offspring on its own [128]. Endothelium-dependent relaxation to acetylcholine was observed to be impaired in mesenteric arteries from 80- and 180-day-old female offspring of obese dams [100]. Samuelsson et al. [129] identified an enhanced response to sodium nitroprusside, an enhanced pressor response to phenylephrine and reduced baroreflex sensitivity in the 30- and 90-day-old offspring of obese dams. Notably, hypertension could be abolished with α- and β-adrenergic blockade. Payen et al. [127] reported that the vascular dysfunction in the offspring of obese dams was associated with altered DNA methylation of CpGs near matrix metalloproteinase, collagen and potassium channel genes in the small mesenteric arteries. Beyond the vasculature, the activity of the sodium-potassium ATPase [130] was significantly reduced, and the expression of renin [129] was increased in the kidneys from offspring of obese dams, which could also influence blood pressure. This suggests that cardiac afterload-dependent effects could be influencing the development of cardiac hypertrophy in adult offspring of obese dams.

## 7. Interventions for the Developmental Origins of Cardiovascular Disease

Physical activity has long been recognized as having important health benefits that can delay or prevent the onset of CVD exposed to GDM and maternal obesity. However, few studies have examined the effects of maternal exercise on the cardiovascular health of the offspring. Given the short gestation length and lifespan of rodents and the ability to carefully control the timing of the exercise intervention, model systems are beneficial for investigating whether maternal exercise can mitigate the adverse effects of maternal obesity and GDM on the offspring.

Several groups have shown that enforced maternal treadmill exercise or voluntary wheel running had significant effects on the metabolic health of male and female offspring [131,132,133,134,135,136]. Using voluntary wheel running, maternal exercise improved glucose tolerance in both mouse and rat offspring, compared to the offspring of sedentary controls [131,132]. In offspring of high-fat diet fed dams, treadmill exercise improved the glucose tolerance of both male and female offspring [134,135,136]. Moreover, maternal exercise also attenuated weight gain and adiposity in 3-month-old offspring fed a high-fat and high-sucrose diet [133]. In general, the most significant effects are observed when maternal exercise begins before mating and is continued throughout pregnancy.

By contrast, only a few studies have been conducted that examined the effect of exercise during a pregnancy characterized by diabetes or maternal obesity on the offspring heart and cardiovascular system. In the offspring of obese dams, a 5 day/week 20 min treadmill exercise intervention prevented maternal obesity-induced cardiac hypertrophy and cardiomyocyte size in 8-week-old offspring [137]. In addition, maternal exercise attenuated maternal obesity-induced left ventricular systolic dysfunction. This was related to the increased expression of troponin, tropomyosin and SERCA2 that are involved in calcium transport and sympathetic-activated inotropic responses. The maternal exercise intervention, however, did not prevent hypertension in the offspring. Using voluntary wheel exercise beginning one week before as well as throughout pregnancy and lactation, Boonpattrawong et al. [138] showed that maternal exercise prevented the adverse effects of maternal obesity on offspring vascular endothelial function. Exercise by obese dams improved thoracic aorta endothelial-dependent vasodilation in adult male offspring fed a postnatal high-fat diet, but not offspring fed the control diet. In addition, serum nitrate and nitrite concentrations were increased in the postnatal high-fat fed offspring of obese dams that performed exercise. Endothelial cells from the offspring of obese dams that exercised had lower expression of genes involved in vasoconstriction, oxidative stress resistance and inflammation compared to the offspring of sedentary obese dams [138]. One study examined diabetes during pregnancy and showed that maternal voluntary wheel running lowered the incidence of congenital heart defects in the offspring [139].

With respect to clinical studies, a recent systematic review examined the effect of exercise during pregnancy on offspring cardiometabolic health outcomes. The included studies were a mixture of observational and randomized controlled trials. While maternal exercise during pregnancy is safe, the systematic review concluded that there was insufficient evidence showing an effect of exercise during pregnancy on offspring metabolic health [140]. However, in this review, high-quality evidence was lacking since there was variability in the type and duration of exercise in these studies; many studies combined exercise with dietary changes and few evaluated health outcomes beyond birth weight in the offspring. Therefore, longer follow-up of studies and analysis of impacts on the cardiovascular system are needed.

Less is known about how pharmaceutical agents or supplements that are used to manage GDM affect the offspring heart. We have recently reviewed this literature [141], and though several studies have examined the effects on the postnatal metabolic health of the offspring, effects on the offspring heart of these different therapies have not been evaluated. Recent work by Cole et al. investigated whether postnatal supplementation with berberine, a glucose-lowering natural health product, attenuated the risk of metabolic syndrome in offspring exposed to GDM [142]. Wild-type C57BL/6 female mice were fed the low-fat or high-fat-and-sucrose diets for 6 weeks prior to breeding and throughout pregnancy and lactation. The offspring were then assigned to postnatal low-fat or high-fat-and-sucrose or the high-fat-and-sucrose diet supplemented with berberine (160 mg/kg/day) at weaning for 12 weeks. Compared to GDM offspring fed a postnatal high-fat-and-sucrose diet, the GDM offspring that received berberine in the high-fat-and-sucrose diet had a lower body weight, increased pancreatic islet glucose-stimulated insulin secretion and improved systolic and diastolic heart function [142]. Berberine could mitigate GDM-induced cardiomyocyte mitochondrial dysfunction through increasing the expression of cardiac enzymes involved in fatty acid uptake and oxidation as well as increasing cardiolipin mass and levels of tetra-linoleoyl cardiolipin, which is essential for respiratory chain activity and supercomplex formation, in the offspring of GDM dams [143]. Berberine has also been shown to exhibit other beneficial cardiometabolic effects, including lipid lowering and anti-inflammatory effects [144,145]. Berberine has been shown to reduce serum LDL cholesterol levels by stabilizing the LDL receptor mRNA, which is a different mechanism of cholesterol lowering achieved by statins and red yeast rice [146]. Therefore, maternal administration of natural compounds could also protect offspring from cardiovascular dysfunction through direct effects on the heart or by mitigating GDM-induced obesity or other cardiometabolic conditions.

## 8. Conclusions

Recent studies using rodent models in a well-controlled environment, summarized herein, point to the important influence that diabetes during pregnancy and maternal obesity have on the cardiovascular system of the offspring to influence CVD development. On the other hand, human studies examining the intergenerational effects of diabetes during pregnancy and maternal obesity on cardiovascular end-points in the offspring at both the population level and in prospective cohort studies are lacking. While the emerging rodent studies have identified a number of potential mechanisms, including, prominently, cardiac mitochondrial dysfunction, more studies are needed to examine the longitudinal changes in the epigenome, transcriptome, proteome and metabolome to determine the dynamics of CVD pathology in the context of DOHaD. In addition, more research targeting these mechanisms as potential treatment avenues, as well as examining the effects of GDM therapeutics on the long-term cardiovascular health of the offspring, should be conducted. Future research directions related to therapeutic interventions should focus on preventing cardiometabolic disease development in offspring exposed to GDM at the earliest possible timepoint, including fetal life.
